# Reticulate phylogeny of gastropod-shell-breeding cichlids from Lake Tanganyika – the result of repeated introgressive hybridization

**DOI:** 10.1186/1471-2148-7-7

**Published:** 2007-01-25

**Authors:** Stephan Koblmüller, Nina Duftner, Kristina M Sefc, Mitsuto Aibara, Martina Stipacek, Michel Blanc, Bernd Egger, Christian Sturmbauer

**Affiliations:** 1 Department of Zoology, University of Graz, Universitätsplatz 2, 8010 Graz, Austria; 2 Section of Integrative Biology, University of Texas at Austin,1 University Station, #C0930, Austin, TX 78712, USA; 3 Graduate School of Bioscience and Biotechnology, Tokyo Institute of Technology, B21-4259, Nagatsuta-cho, Midori-ku, Yokohama 226-8501, Japan

## Abstract

**Background:**

The tribe Lamprologini is the major substrate breeding lineage of Lake Tanganyika's cichlid species flock. Among several different life history strategies found in lamprologines, the adaptation to live and breed in empty gastropod shells is probably the most peculiar. Although shell-breeding arose several times in the evolutionary history of the lamprologines, all obligatory and most facultative shell-breeders belong to the so called "ossified group", a monophyletic lineage within the lamprologine cichlids. Since their distinctive life style enables these species to live and breed in closest vicinity, we hypothesized that these cichlids might be particularly prone to accidental hybridization, and that introgression might have affected the evolutionary history of this cichlid lineage.

**Results:**

Our analyses revealed discrepancies between phylogenetic hypotheses based on mitochondrial and nuclear (AFLP) data. While the nuclear phylogeny was congruent with morphological, behavioral and ecological characteristics, several species – usually highly specialized shell-breeders – were placed at contradicting positions in the mitochondrial phylogeny. The discordant phylogenies strongly suggest repeated incidents of introgressive hybridization between several distantly related shell-breeding species, which reticulated the phylogeny of this group of cichlids. Long interior branches and high bootstrap support for many interior nodes in the mitochondrial phylogeny argue against a major effect of ancient incomplete lineage sorting on the phylogenetic reconstruction. Moreover, we provide morphological and genetic (mtDNA and microsatellites) evidence for ongoing hybridization among distantly related shell-breeders. In these cases, the territorial males of the inferred paternal species are too large to enter the shells of their mate, such that they have to release their sperm over the entrance of the shell to fertilize the eggs. With sperm dispersal by water currents and wave action, trans-specific fertilization of clutches in neighboring shells seem inevitable, when post-zygotic isolation is incomplete.

**Conclusion:**

From the direct observation of hybrids we conclude that hybridization between distantly related gastropod-shell-breeding cichlids of Lake Tanganyika follows inevitably from their ecological specialization. Moreover, the observed incongruence between mtDNA and nuclear multilocus phylogeny suggests that repeated hybridization events among quite distantly related taxa affected the diversification of this group, and introduced reticulation into their phylogeny.

## Background

With an age of 9–12 million years, Lake Tanganyika is the oldest of the three East African Great Lakes and harbors the morphologically, behaviorally and ecologically most diverse cichlid species flock, whose ancestors colonized the swampy and shallow ecosystem of the emerging lake shortly after its formation [1]. The 250 or more cichlid species of Lake Tanganyika have been grouped into a number of mostly endemic tribes, among which the tribe Lamprologini [2] is the most species-rich with >80 species. The Lamprologini are the only substrate-breeding endemics in Lake Tanganyika; nearly all of the remaining species are maternal or biparental mouthbrooders. Several of the Lamprologini developed a distinctive life style, in that they live and breed in empty shells of the gastropod species *Neothauma tanganyicense*, and sometimes *Pila ovata*, *Paramelania *spp. and *Lavigeria *spp. [3,4]. Shell-breeding represents a highly successful evolutionary strategy, and although the behavior arose multiple times during the radiation of the lamprologines [5], most shell-breeding species are members of the "ossified group", a monophyletic group of 28 species characterized by a sesamoid bone within the labial filament [6]. The "ossified group" includes both the smallest (3.5 cm) and the largest lamprologine (>30 cm); in shell-breeding species, either both sexes are sufficiently small to fit in gastropod-shells, or the sexes display extreme size dimorphism with dwarf females and males too large to enter the shells [7].

**Figure 1:**
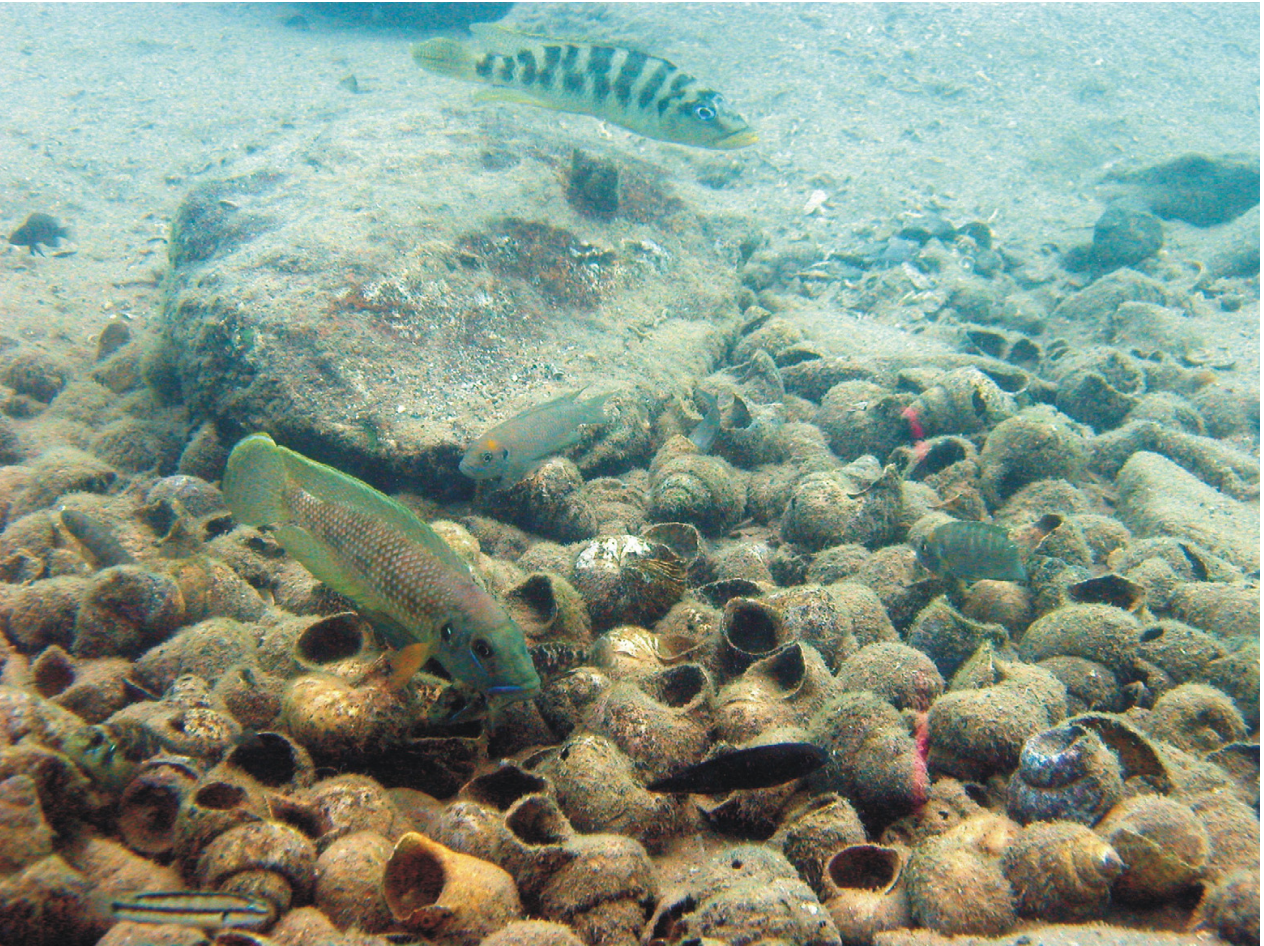
**A typical shell-nest constructed by large *Lamprologus callipterus *males**. These aggregations attract different species of obligatory and facultative gastropod-shell-breeders, which consequently live and breed in closest vicinity. The photograph shows individuals of *L. callipterus, N. calliurus, N. fasciatus *and two facultative "non-ossified group" shell-dwellers – *Telmatochromis temporalis *and *T. vittatus*.

Most shell-breeding species utilize shell-beds aggregated by wave action or individual shells scattered on the lake floor, but males of one species – *Lamprologus callipterus *– collect and pile shells to nests [3]. Some of these shells may already be inhabited by another shell breeder, which thus gets transferred into the *L. callipterus *nest. Additionally, shell-breeders often colonize these nests actively, such that shell nests and natural shell aggregations often house a densely packed multi-species community of individuals living and breeding in closest vicinity (Fig. 1). Given that large males spawn openly over the mouth of their mate's shell, this concentration of breeding pairs of different species sets the stage for accidental hybridization and hybridization associated with the employment of alternative reproductive strategies [7].

In order to obtain a better understanding of the evolution and pathways of diversification of shell-breeding lamprologine cichlids, we aimed to reconstruct the phylogeny of the "ossified group" of lamprologines by means of sequences of the entire mitochondrial NADH dehydrogenase subunit 2 gene (ND2) and a set of AFLP markers, and investigate the role of past and ongoing hybridization in the diversification of this group of fishes. Morphological data were incorporated in the analyses to infer hybridization partners.

## Results and discussion

### Hybrid speciation reticulates the phylogeny of the "ossified group" of lamprologines

**Figure 2:**
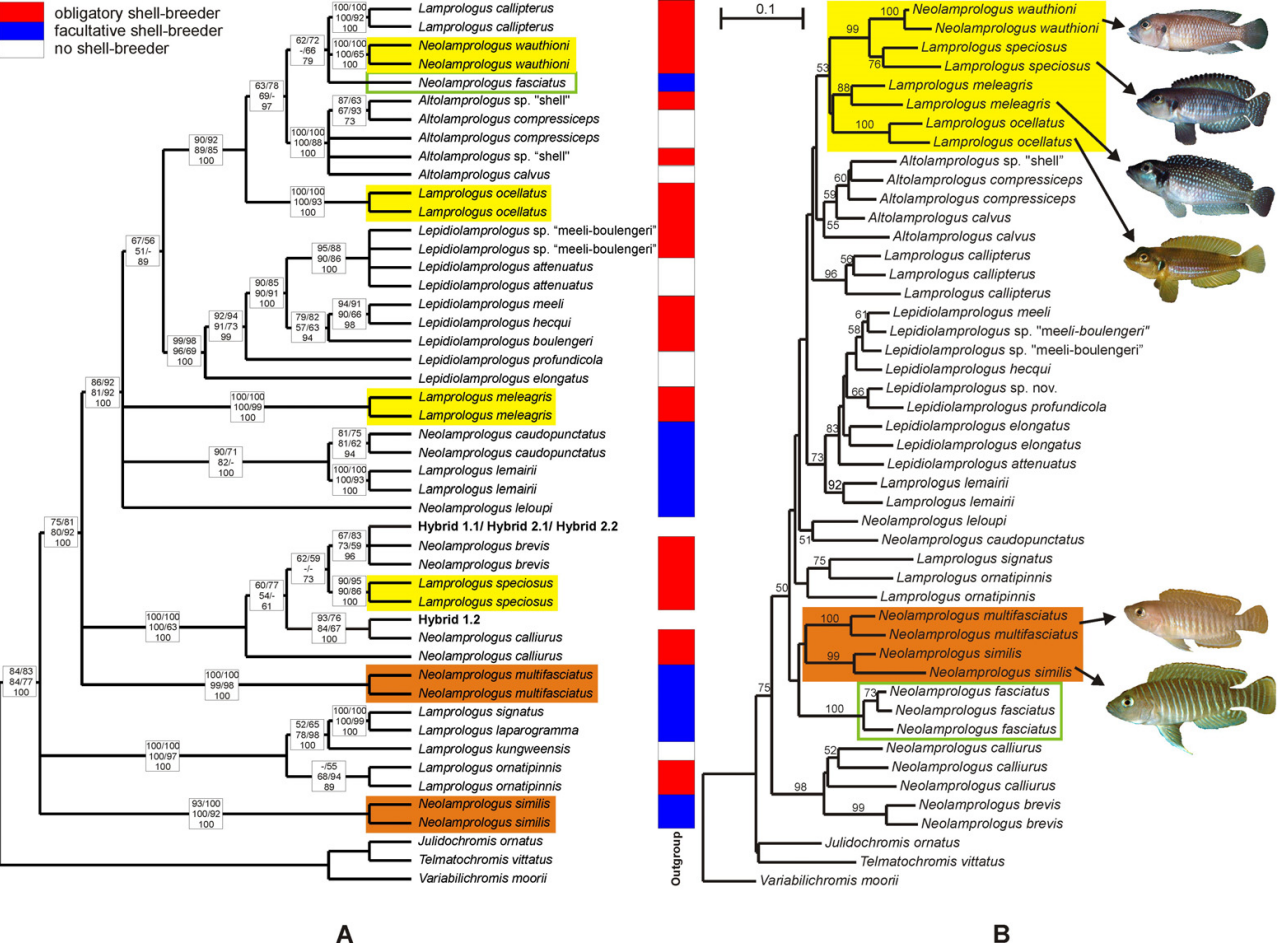
**Phylogenetic relationships among the "ossified group" lamprologines**. The incongruency between current genus assignments and phylogenetic relationships and the consequent need of a taxonomic revision has been addressed previously [5, 6, 8]. Here, the nomenclature of species in the genus *Lepidiolamprologus *follows [8] (A) Strict consensus tree of the results obtained from maximum parsimony analysis (MP; 48 most parsimonious trees; tree length, 1390 evolutionary steps; CI excluding uninformative characters, 0.6014; RI, 0.7503; RC, 0.4513), neighbour-joining (NJ), maximum likelihood (ML) and Bayesian inference (BI) based on ND2 sequence data of 48 taxa, representing 27 species of the "ossified group" of lamprologines, four putative hybrid specimens and three outgroup taxa. Boxes on the branches contain bootstrap values obtained from NJ and MP (upper line), ML-bootstrap and quartet puzzling values (middle line), and Bayesian posterior probabilities (bottom line). Only values higher than 50 are shown. Colored bars code for different breeding behaviors. (B) NJ tree based on Nei and Li's distances of the AFLP data of 47 taxa, representing 26 species of the "ossified group" of lamprologines and three outgroup taxa. Bootstrap values > 50 are shown above the branches. The photographs illustrate the phenotypic similarity between *Lamprologus meleagris, L. ocellatus, L. speciosus *and *Neolamprologus wauthioni*, and between *N. similis *and *N. multifasciatus*. Colored boxes mark eco-morphologically similar taxa with incongruent positions in the mitochondrial and nuclear phylogenies, as well as the disparate placement of *N. fasciatus*.

**Figure 3:**
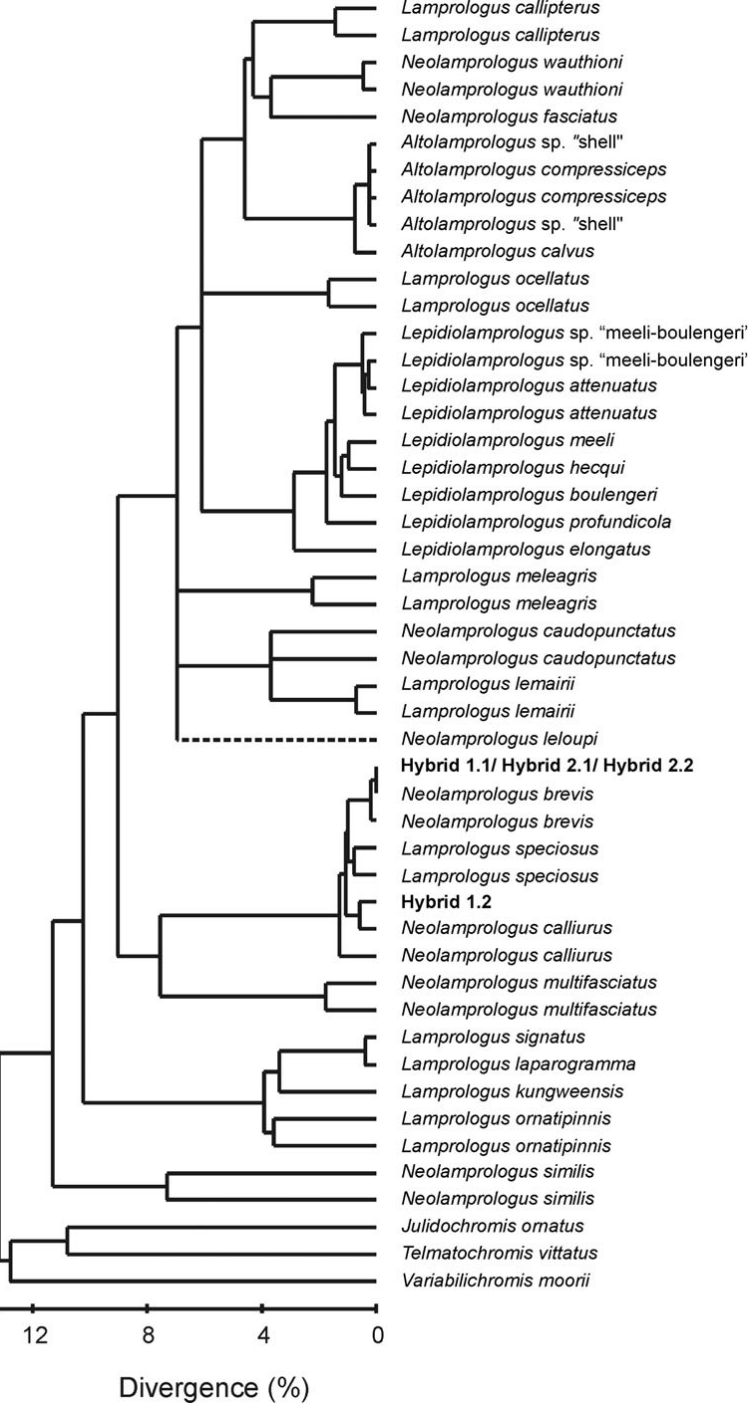
**Linearized tree**. It is based upon a 1047 bp segment of the mitochondrial ND2 gene applying the substitution model TrN+Γ [46]. After performing a branch length test [49], *Neolamprologus leloupi *was excluded from the analysis due to a significantly deviating rate of base substitution and subsequently added to the phylogeny according to the results of NJ, MP, ML and Bayesian analysis. The distance values in the scale below the phylogenetic tree correspond to the observed mean sequence divergence using the substitution model TrN+Γ.

Reticulate evolution became first apparent in phylogenetic reconstructions based on 1047 bp of the mitochondrial ND2 gene. The phylogeny of the ossified-group lamprologines (Fig. 2A) is largely congruent with previous reconstructions based on different taxon sampling [5,8], subdividing the "ossified group" into 4 – 5 clades depending on the tree-building algorithm used. A linearized tree analysis suggests that the majority of clades arose at a divergence level of about 7% (TrN+Γ distances; Fig. 3), which corresponds to major cladogenesis events in other Lake Tanganyika cichlid lineages [9-11] and platythelphusid crabs [12]. Their diversification may thus have been induced by the same environmental factor, most likely by a substantial drop of the lake level during a period of aridification in eastern Africa about 2.5 to 3 million years ago [13].

Despite the strength of the phylogenetic signal and the high resolution of reconstructed species relationships, the mtDNA phylogeny contains several groupings in striking conflict with morphological, ecological and behavioral similarities. Remarkably, all inconsistencies involve gastropod shell breeders. The first pair of eco-morphologically and behaviorally highly similar species resolved in different clades on the mitochondrial tree is *Neolamprologus similis *and *N. multifasciatus *(Fig. 2A). Males and females of both species are sufficiently small to enter gastropod shells – *N. multifasciatus *is the smallest Tanganyikan cichlid known to date (3.5 cm)- and both species are facultative shell-breeders, which arrange their nests by excavating sand craters around small accumulations of empty shells and live in family groups. The second group of morphologically and behaviorally similar species separated in the mitochondrial phylogeny includes *Lamprologus ocellatus*, *L. meleagris*, *L. speciosus *and *N. wauthioni*. These species are sexually monomorphic (see Table 1 for more information), small-bodied obligatory shell-breeders with a maximum size of about 6 cm that prefer areas with low densities of empty gastropod shells. In each species, the males defend territories and hold harems of two to five females for whom they bury shells in a way that only the opening of the shell remains accessible to a resident female [14]. *L. meleagris *occurs sympatrically with *N. wauthioni *in the north, and with *L. speciosus *in the south of its distribution range. Despite their morphological and behavioral similarity, each of these four species clusters with different, eco-morphologically very dissimilar, species (Fig. 2A). Although contrasting with phenotypic data, the mitochondrial tree topology receives high bootstrap support, and a monophyletic clade of *L. speciosus*, *L. meleagris*, *L. ocellatus *and *N. wauthioni *requires at least 56 additional steps in a MP phylogeny (P < 0.001; Δ -ln L = 206.84, P < 0.001). Forcing *N. similis *and *N. multifasciatus *into a monophyletic clade did not significantly increase tree length (P > 0.05; Δ -ln L = 24.43, P > 0.05), but bootstrap support for the non-sister group relationship between *N. multifasciatus *and *N. similis *was high.

**Table 1:** Life history traits of the species assigned to the „ossified group" of lamprologines.

**species**	**Shell-breeding**	**parental care**	**mating system**
*Altolamprologus calvus*	no	MG	monogamy?
*Altolamprologus compressiceps*	no	MG	harem
*Altolamprologus *sp. "shell"	obligatory	?	?
*Lamprologus callipterus*	obligatory	MG	harem
*Lamprologus kungweensis*	no	MG	monogamy
*Lamprologus laparogramma*	facultative	MG	monogamy
*Lamprologus lemairii*	facultative	MG	harem
*Lamprologus meleagris*	obligatory	MG	harem
*Lamprologus ocellatus*	obligatory	MG	harem
*Lamprologus ornatipinnis*	obligatory	MG	harem
*Lamprologus signatus*	facultative	MG	monogamy
*Lamprologus speciosus*	obligatory	MG	harem
*Lepdiolamprologus attenuatus*	facultative*	BG	mono(bi)gamy
*Lepidiolamprologus boulengeri*	obligatory	MG	monogamy?
*Lepidiolamprologus elongatus*	no	BG	mono(bi)gamy
*Lepidiolamprologus hecqui*	obligatory	MG	?
*Lepidiolamprologus kendalli*^+^	no	BG	monogamy?
*Lepidiolamprologus meeli*	obligatory	MG	harem
*Lepidiolamprologus nkambae*^+^	no	BG	monogamy?
*Lepidiolamprologus pleuromaculatus*^+^	facultative	MG	harem
*Lepidiolamprologus profundicola*	no	MG	harem
*Lepidiolamprologus *sp. "meeli-boulengeri"	obligatory	MG	harem
*Lepidiolamprologus *sp. nov	no	?	?
*Neolamprologus brevis*	obligatory	MG	harem
*Neolamprologus calliurus*	obligatory	MG	harem
*Neolamprologus caudopunctatus*	facultative	BG	monogamy
*Neolamprologus fasciatus*	facultative	MG	harem
*Neolamprologus leloupi*	facultative	BG	monogamy
*Neolamprologus multifasciatus*	facultative	MG	monogamy?
*Neolamprologus similis*	facultative	MG	monogamy?
*Neolamprologus variostigma*^+^	no	?	?
*Neolamprologus wauthioni*	obligatory	MG	harem

*Note*: In all species males grow slightly larger than females, but there are only five species – *Lamprologus callipterus*, *Lamprologus lemairii, Lamprologus ornatipinnis*, *Neolamprologus calliurus*, *Neolamprologus fasciatus *– exhibiting extreme sexual size dimorphism with males growing to at least the double size of females.

MG, maternal guarding; BG, biparental guarding.

^+^, species is not included in this study.

*, *Lepidiolamprologus attenuatus *is too large to enter empty gastropod-shells. Thus, this species occasionally deposits its eggs on the outside of the shell; after hatching fry is transferred into the shell.

Information on the species listed is based on [3, 4, 14, 63–65].

In contrast, species relationships inferred from a set of 199 polymorphic AFLP markers were consistent with predictions based on phenotypic trait similarities, as each of the two groups of eco-morphologically similar species was resolved as a monophyletic clade (Fig. 2B). Disparate mitochondrial and nuclear reconstructions were also obtained for the phylogenetic position of another shell-breeding species: *Neolamprologus fasciatus *clustered with *L. callipterus *and *N. wauthioni *in the mtDNA-phylogeny, but was placed ancestral to *N. multifasciatus *and *N. similis *by AFLP analyses. A constrained mitochondrial tree congruent with the nuclear placement of *N. fasciatus *would require significantly more evolutionary steps under both parsimony and likelihood criteria (at least 22 steps in MP, P < 0.001; Δ -ln L = 97.08, P < 0.001 in ML). Although the current data allow only limited inference of the phylogenetic history of *N. fasciatus*, we can definitely rule out a close affinity to the genus *Altolamprologus*, which has been suggested due to superficial morphological similarities [14].

Ancient hybridization and ancient incomplete lineage sorting are alternative explanations for the observed discrepancy between the mitochondrial phylogeny and relationships predicted by nuclear markers, phenotypes and behavior. Mismatches among gene trees can result from the differential assortment of ancestral polymorphisms, when intervals between successive branching events are too short for lineage sorting to be completed within each branch prior to the next split [15,16]. This phenomenon occurs, for example, when taxa speciate rapidly during adaptive radiation, and has been repeatedly reported from the cichlid species flocks in the East African Great Lakes [15,17-20]. In the mitochondrial phylogeny of the shell-breeding Lamprologini, the taxa affected by inconsistent mitochondrial and nuclear tree topologies (*L. speciosus*, *L. meleagris*, *L. ocellatus *and *N. wauthioni*; and *N. multifasciatus *and *N. similis*) have their most recent common ancestor near or at the base of the tree. Long interior branches and bootstrap support for many interior nodes in the mitochondrial phylogeny do not indicate a rapid burst of cladogenesis, such that there may be no major effect of ancient incomplete lineage sorting on the phylogenetic reconstruction.

Nuclear loci coalesce more slowly [21], and are therefore subjected to lineage sorting incongruence over longer periods. The combination of multiple independent loci, as implemented by the AFLP technique, is expected to overcome the problem of idiosyncratic lineage sorting at individual loci [22-24]. Additionally, the species relationships suggested by our AFLP data are supported by phenotypic and behavioral similarities, which makes it unlikely that the AFLP clades result from stochastic lineage sorting rather than reflecting actual relationships.

**Figure 4:**
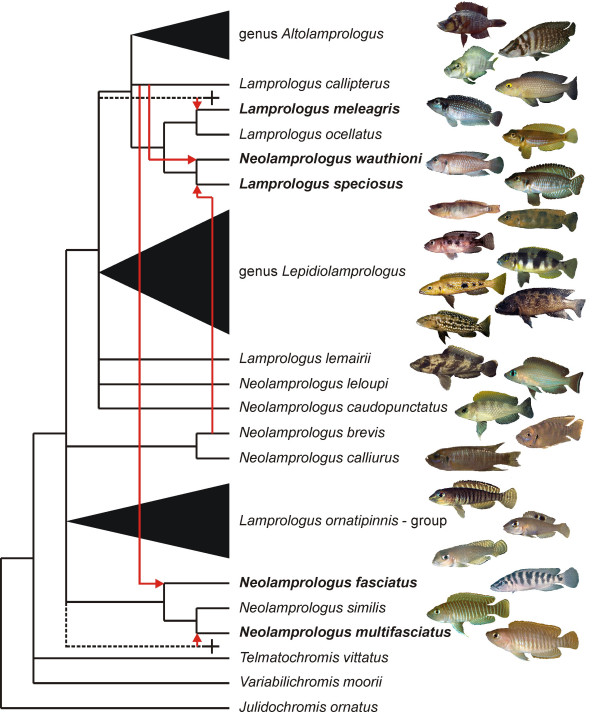
**Reticulation of the species phylogeny by hybrid speciation**. A strict consensus of mitochondrial and nuclear phylogenies was constructed from the subset of species that was assumed to have undergone bifurcating speciation. The inferred hybrid species (indicated in bold) were added according to their positions in the nuclear phylogeny. Stippled branches indicate hypothesized, now extinct lineages; red arrows indicate the direction of introgression of the mitochondrial genome into the hybrid species. Photographs show the large degree of morphological diversity in the ossified-group lamprologines.

We contend that the incongruence between the mitochondrial phylogeny and multiple nuclear markers, phenotypes and behavior is best explained by ancient introgressive hybridization events. Furthermore, it is possible that hybridization was directly associated with the speciation of *L. meleagris*, *N. wauthioni*, *L. speciosus*, and *N. multifasciatus *(Fig. 4), and perhaps also *N. fasciatus*. Species status according to the biological species concept [25] of the presumed hybrid taxa is supported by sympatric occurrences of *L. meleagris *with *L. speciosus *in the south and *N. wauthioni *in the north of its distribution range, and the lake-wide, continuous distribution of *N. fasciatus*.

Although published mitochondrial phylogenies of the mouthbrooding tribes encompass representative species samples [[9-11,26,27], and unpublished data), striking incongruencies with behavioral, ecological and morphological data were detected only in the tribes Tropheini [26,28,29] and Eretmodini [30]. In the tropheine genus *Petrochromi*s, the incongruence was explained by parallel evolution of distinct eco-morphotypes [26], whereas disparate mitochondrial placements of color morphs and species in the tropheine genus *Tropheus *[28,29] and in the tribe Eretmodini [20] were attributed to introgressive hybridization after secondary admixis. In contrast, diversifying hybridization has been proposed in studies of the substrate breeding Lamprologini, including a role of introgressive hybridization in the speciation of *Neolamprologus marunguensis *[31] and of *Lepidiolamprologus nkambae*, a non-shell-breeding member of the "ossified group" of lamprologines [8]. Our results with the shell-breeding lamprologines provide a further example of potential evolutionary consequences of introgressive hybridization in another guild of lamprologine cichlids.

In two instances, species were resolved non-monophyletically. *Altolamprologus calvus *was paraphyletic in relation to *A. compressiceps *in the AFLP tree, and *Lepidiolamprologus *sp. "meeli-boulengeri" was paraphyletic with respect to *L. meeli *in the AFLP tree, while the two taxa were placed in different clades by the mitochondrial sequences. The taxonomy of these species is not well resolved, in that populations with intermediate phenotypes and phenotypic divergence across geographic distances have not yet been adequately addressed (Schelly, pers. comm.). The here identified incongruence between the phylogenetic and current taxonomic resolution must not be dismissed as a possible consequence of persistent ancestral polymorphism in the genetic data, but pinpoints a need for more detailed taxonomic and molecular genetic work on these taxa.

### Ongoing hybridization among "ossified group" lamprologines

**Figure 5:**
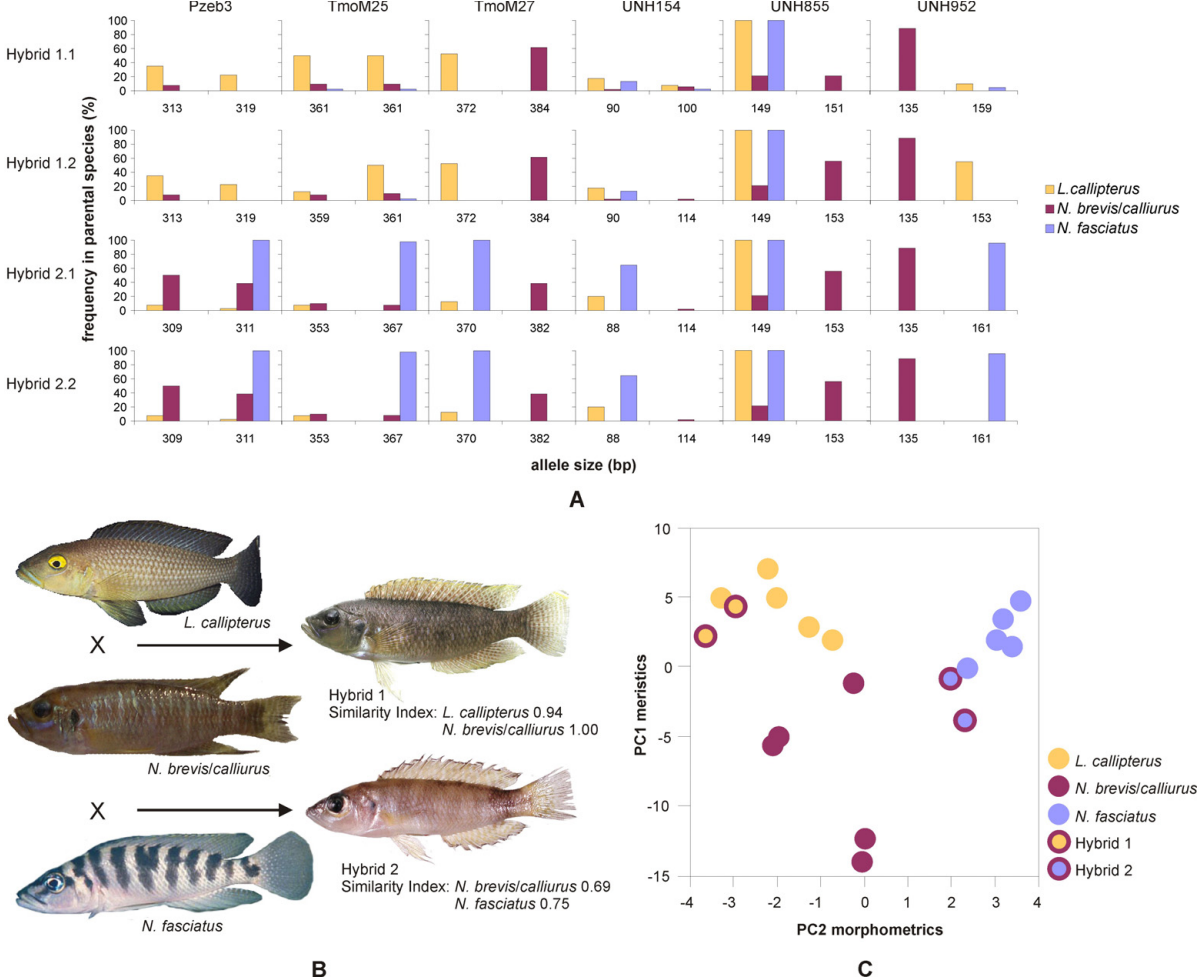
**Genetic and morphological identification of hybrid parents**. Hybrid 1.1 and hybrid 1.2 result from crosses between *L. callipterus *and a member of the clade encompassing *N. brevis *and *N. calliurus*, hybrid 2.1 and hybrid 2.2 from hybridization between *N. fasciatus *and *N. brevis*/*calliurus*. (A) Allelic composition of the four hybrid specimens at six microsatellite loci. Bars indicate alleles detected in hybrid individuals with bar height proportional to the frequencies of these alleles in each of the putative parental species. (B) Pictures of the hybrid specimens and their parental species and similarity indices based on 13 qualitative morphological characters demonstrate the intermediate phenotype of the hybrids. Hybrid individuals originating from the same species pair do not differ phenotypically, and only one individual of each hybrid type is shown. (C) Principal component analysis based upon 13 morphometric and 8 meristic measurements place hybrid individuals with the inferred paternal species.

Evidence for past hybridization affecting the evolutionary history of the "ossified group" of lamprologines obviously raises the question whether there is also evidence for ongoing hybridization among shell-breeding cichlids. Indeed, within a small area of suitable habitat containing several *L. callipterus *nests in southern Lake Tanganyika (near Wonzye, Zambia), we collected four specimens that could not be identified to species level but were clearly lamprologine cichlids. Although lamprologines hybridize readily in captivity, this is the first proof of viable natural hybrids in Lake Tanganyika. Mitochondrial sequences determined that the mothers of the hybrids were members of the *N. brevis*/*N. calliurus *– clade (Fig. 2A). Hybrid phenotypes and data from six microsatellite loci suggested that two of the hybrids were sired by *L. callipterus *and the other two by *N. fasciatus *(Fig. 5A). Morphological similarity indices (Fig. 5B), and a principal component analysis of morphometric and meristic measurements (Fig. 5C) further supported the phenotypically and genetically inferred identity of the parental species. Furthermore, territorial males of the two putative paternal species *L. callipterus *and *N. fasciatus *are too large to enter empty shells (see Fig. 1), and release their sperm over the entrance of their mate's shell to fertilize her eggs. Dispersion of sperm into adjacent shells by water currents and wave action can bypass prezygotic isolation between species sharing a shell-bed, and result in trans-specific fertilization and hybridization, when post-zygotic isolation is incomplete.

Within the lamprologines, the inferred hybridization partners are only distantly related, as the split between the mitochondrial lineages including *N. brevis*/*calliurus *and the lineage containing *L. callipterus *and *N. fasciatus *is estimated as 3.80 (± 1.33) – 4.58 (± 1.60) million years before presence (TrN+Γ distance, 9.08 ± 1.33%; gamma-corrected amino acid distance, 4.44 ± 1.55%) based on a molecular clock for the ND2 gene of cichlid fish [11]. The two *N. fasciatus *hybrids and one of the *L. callipterus *hybrids shared a mitochondrial haplotype, whereas a different haplotype was detected in the second *L. callipterus *hybrid (Fig. 1). Hence, the four hybrid individuals represent at least three independent hybridization events between the genetically and morphologically highly divergent species, suggesting that hybridization among shell-breeding species is not uncommon. Introgressive hybridization is commonly associated with loss of diversity through extinction or homogenization of species [32-34], and the maintenance of species integrity in the face of hybridization suggests that introgression between lamprologine species is constrained by low hybrid fitness. Accidental hybridization among shell-breeders is independent of prezygotic species barriers and unresponsive to reinforcement of pre-mating isolation, such that hybridization rates may not decrease over long divergence times despite possible costs through reduced hybrid fitness.

## Conclusion

Compared to plants [35], the role of hybridization in speciation and diversification in animals has been demonstrated in only a few examples [36] including several cichlid species [8,31,37,38]. Our study suggests that shell-breeding lamprologine cichlids experience considerable rates of hybridization without suffering significant erosion of phenotypic diversity. The fixation of introgressed mitochondrial haplotypes reticulated the evolution of this group. Although our data do not provide direct proof of speciation by hybridization, they are consistent with the hypothesis that hybridization might have contributed to the diversification of gastropod shell-breeding cichlids. The high degree of morphological differentiation among lamprologine species entails elevated levels of functional diversity and a strong potential for transgressive segregation in hybrid populations, a condition that allows for the occasional emergence of a successful novel trait combination [38-40]. Stabilization of a recombinant population requires reproductive isolation from the parental species, which has been shown to ensue from ecological separation from both parent species in several cases of hybrid speciation [39]. During the evolutionary history of Lake Tanganyika cichlids, periodical habitat changes associated with lake level fluctuations may have occasionally promoted the stabilization of hybrid populations by providing novel ecological niches, and by precipitating displacements and splits of populations [41], such that geographic separation may have added to or even substituted for ecologically mediated isolation.

## Methods

### Sampling

Representatives of 31 lamprologine cichlid species, including the outgroup taxa *Variablichromis moorii*, *Telmatochromis vittatus *and *Julidochromis ornatus*, were collected during several sampling trips to Lake Tanganyika from 1992 to 2004 or obtained via the aquarium trade. Fin clips were taken from fresh specimens and preserved in 96% ethanol. Voucher specimens are available from the authors.

### mtDNA

We analyzed 1047 bp of the entire mitochondrial ND2 gene for 69 individuals, representing 30 sampled species and four putative hybrids. When available, previously published sequences were used [1,8]. Total DNA-extraction, polymerase chain reaction (PCR) and chain termination sequencing followed standard protocols [9,42]. For both, PCR and chain termination reaction sequencing, we used the primers MET, ND2.2A, TRP [43] and ND2.T-R [11]. Sequences were visualized on an ABI 3100 Sequencer (Applied Biosystems). All sequences are available from GenBank under the accession numbers listed in Additional File 1.

Alignment of DNA sequences was performed using the Sequence Navigator software (Applied Biosystems). Throughout the presented phylogenetic analyses, hierarchical likelihood ratio test statistics were calculated using the program Modeltest 3.06 [44] to evaluate appropriate models of molecular evolution for model based tree reconstructions, and phylogenetic reconstruction based on NJ, ML and MP criteria were calculated with the PAUP* program package (version 4.0b5) [45]. In a first step, a NJ tree including all 69 taxa {substitution model TrN+I+Δ, [46]; proportion of invariable sites (I), 0.4147; gamma shape parameter (α), 0.9162} was used to select a representative subset of 48 taxa to minimize computation time for subsequent ML and maximum parsimony (MP) analysis. The reduced data set was then used for NJ and ML analyses (substitution model TrN+I+ Δ; I, 0.4083; α, 0.8659; base frequencies: A, 0.2530; C, 0.3531; G, 0.1308; T, 0.2630), and for MP tree searches with transitions at third codon positions weighted 3:1 with respect to transversions, based on the estimated transition/transversion (TI/TV) ratio of 2.9627. Transversions at third codon positions of fourfold degenerate amino acids were weighted 2:1 with respect to transitions, according to the estimated TI/TV ratio of 2.1140. Synonymous transitions at first positions of leucine codons were treated like transitions at third codon positions. Bootstrapping (1000 pseudo-replicates for NJ and MP; 100 pseudo-replicates for ML) and quartet puzzling [47] (25 000 random quartets) were applied to estimate support of the obtained topologies. Phylogenetic relationships were also estimated by a Bayesian method of phylogenetic inference using MRBAYES 3.0b4 [48]. Posterior probabilities were obtained from a 1,000,000 generation Metropolis-coupled Markov chain Monte Carlo simulation (four chains; chain temperature, 0.2) with parameters estimated from the data set. Trees were sampled every hundred generations and the first 10% of all trees were excluded as burn-in to allow likelihood values to reach stationary.

To derive a relative dating of diversification events in the "ossified group" of lamprologines, we computed a linearized tree, using the program LINTRE [49]. To test for constancy of the rate of base substitution among all taxa we performed a branch length test implemented in the program LINTRE based on TrN+ Δ distances (α = 0.2841). *Neolamprologus leloupi *showed a significantly deviating substitution rate at the 1% level and was therefore excluded from the calculation of a clock-constrained tree. Mean and standard deviation of average pairwise TrN+ Δ distances were calculated to achieve a relative dating of diversification events. Since no fossil record is available as calibration point for the "ossified group" of lamprologines, a molecular clock for the cichlid ND2 gene based upon gamma corrected amino acid distances [11], calculated with the program TREE-PUZZLE 5.0 [50], was applied to obtain absolute datings for cladogenesis events.

The significance of differences between alternative tree topologies was evaluated by Kishino-Hasegawa tests [51] and Shimodeira-Hasegawa tests [52] under maximum parsimony and likelihood criteria, respectively.

### AFLPs

For AFLP fingerprinting, whole genomic DNA was extracted from 47 specimens representing 25 species of the "ossified group" of lamprologines and the three outgroup species *Julidochromis ornatus*, *Telmatochromis vittatus *and *Variabilichromis moorii *applying proteinase K digestion followed by protein precipitation with ammonium acetate and ethanol precipitation of DNA. Restriction digestion of 100 ng of genomic DNA was performed in a total volume of 50 μl using 0.5 μl *Mse*I (10 units/μl, New England Biolabs), 0.25 μl *EcoR*I (200 units/μl, New England Biolabs), 5 μl enzyme buffer (10×), 0.5 μl BSA (100×) and high performance liquid chromatography (HPLC) water, and incubation for three hours at 37°C. For ligation of adaptors, 1 μl *EcoR*I-adaptor (50 pmol/μl), 1 μl *Mse*I-adaptor (5 pmol/μl), 1 μl T4 ligase buffer, 0.2 μl T4 DNA ligase and 6.8 μl HPLC water were added to the product of the restriction digestion and incubated over night at 37°C. The ligation products were subsequently diluted to 180 μl using HPLC water. PCR was carried out in two steps as recommended by [53]. Preselective amplifications were performed with 3 μl of the diluted ligation product, 0.4 μl each of *EcoR*I and *Mse*I preselective primers (10 μM), 2 μl 10 × MgCl_2 _buffer, 2 μl 10 × dNTP mix (10 μM) and 0.6 μl *Taq *DNA polymerase (5 units/μl, BioTherm™) in a final volume of 20 μl. The preselective primers consisted of the adaptor primer sequence with a single selective nucleotide at the 3' end (*EcoRI*-pre: A, *MseI*-pre: C). The preselective PCR used the following temperature profile: 2 min at 72°C followed by 20 cycles of 20 sec at 94°C, 30 sec at 56°C, and 2 min at 72°C, then a holding step at 60°C for 30 min. PCR products were diluted 1:10 for selective amplification. The following recipe was used for selective amplifications with primers extending 3 bp beyond the adaptor sequence: 1 μl of the diluted preselective PCR product, 6.3 μl HPLC water, 0.8 μl 10 × dNTP mix (10 μM), 1 μl 10 × MgCl_2 _buffer, 0.4 μl *Taq *DNA polymerase (5 units/μl; BioTherm™), 1 μl selective *Mse*I primer (10 μM) and 1 μl selective *EcoR*I primer (1 μM) labeled with the fluorescent dye FAM. The primer combinations used for selective amplification were: *EcoR*I-ACA/*Mse*I-CAT, *EcoR*I-ACT/*Mse*I-CAT, *EcoR*I-ACT/*Mse*I-CAA, *EcoR*I-ACT/*Mse*I-CAC. The temperature profile for the selective PCR was as follows: 2 min at 94°C followed by 10 cycles with 20 sec at 94°C, 30 sec at annealing temperature, which decreased in each cycle by 1°C from 65°C to 56°C, and 2 min at 72°C. The PCR continued for 25 cycles with 20 sec at 94°C, 30 sec at 56°C, and 2 min at 72°C, followed by a holding step at 60°C for 30 min. All amplifications were performed on a GeneAmp PCR system 9700 (Applied Biosystems). Selective amplification products were visualized on an ABI 3100 automated sequencer (Applied Biosystems) along with an internal size standard (GeneScan-500 ROX, Applied Biosystems).

Raw fragment data were analyzed using GENEMAPPER (version 3.7, Applied Biosystems). Presence or absence of peaks (presumed to represent homologous fragments) was scored by eye (in order to avoid misinterpretations inherent to automated fragment scoring) within a range of 100–500 bp and assembled as a binary (1/0) matrix by GENEMAPPER. In a few cases, fragments were scored as missing data when character states could not be determined unambiguously. Matrices from the different primer combinations were assembled into one data set.

A neighbor joining tree based on pairwise genetic distances [54] obtained from the presence/absence matrix was calculated with the program TREECON 1.3b [55]. Bootstrap values from 1000 pseudo-replicates were used as a standard measure of confidence in the reconstructed tree topology.

### Morphological analysis

Similarity-indices between hybrid individuals and eight possible parental species were calculated based upon the presence or absence of 13 qualitative characters (Additional File 2). Furthermore, a principal component analysis based upon 13 morphometric (Additional File 3) and 8 meristic (Additional File 4) characters [56,57] in the four hybrids and the presumed parental species was performed (n = 5 individuals per species). Since the first principal component of the morphometric dataset proofed to be mainly influenced by the standard length, it was not used for analysis. Thus, we plotted PC1 of the meristic data against PC2 of the morphometric data. Loading factors are depicted in Additional Files 5 and 6.

### Microsatellites

Microsatellite markers were used to support morphological inferences of species identity of the parents of the collected hybrid individuals. The four hybrid individuals, one to three representatives of 15 species of obligatory and facultative shell-breeding lamprologines and populations of the three candidate parent species (as identified by morphological criteria) were genotyped at six microsatellite loci with low intraspecific variation in lamprologine cichlids (Pzeb3 [58], TmoM25, TmoM27 [59], UNH154 [60], UNH855 and UNH952 [61]; see Additional Files 7 and 8). Populations of the three candidate parental species, *Lamprologus callipterus *(n = 19), *Neolamprologus brevis*/*calliurus *(n = 24) and *N. fasciatus *(n = 21) were sampled at the same locality as the hybrid individuals (Wonzye, S 08°43', E 31°08'). Mitochondrial sequences placed all four hybrid individuals in the clade containing *N. brevis *and *N. calliurus*, which are very closely related and often synonymized since sub-adult individuals of these two species cannot be distinguished in the field (Figs. 2, 3, 4).

Total DNA-extraction and amplification of microsatellites followed standard protocols [62]. Fragments were visualized on an ABI 377 automated sequencer (Applied Biosystems) using forward primers labeled with fluorescence dyes FAM, TET or HEX, and the internal size standard Genescan-500 TAMRA (Applied Biosystems).

## Authors' contributions

SK, ND, KMS and CS designed the study and were involved in sampling. SK, ND, MS, MB and BE carried out the molecular work and the analyses, whereas MA was responsible for the morphological data. SK, ND, KMS and CS contributed to the preparation of the manuscript. All authors read and approved the final version.

## Supplementary Material

Additional file 1 Species information, geographic origin and GenBank accession numbers of all taxa used for phylogenetic analysis. Click here for file

Additional file 2 Matrix of characters used for estimating similarity indices between hybrids and candidate parental species. Click here for file

Additional file 3 Morphometric measurements for PCA of hybrids and parental species. Click here for file

Additional file 4 Meristic measurements for PCA of hybrids and parental species. Click here for file

Additional file 5 Factor loadings of morphometric measurements on the first three principal components of *Lamprologus callipterus *(n = 5), *Neolamprologus brevis *(n = 5), *N. fasciatus *(n = 5), hybrid 1 (n = 2) and hybrid 2 (n = 2). Click here for file

Additional file 6 Factor loadings of meristic measurements on the first three principal components of *Lamprologus callipterus *(n = 5), *Neolamprologus brevis *(n = 5), *N. fasciatus *(n = 5), hybrid 1 (n = 2) and hybrid 2 (n = 2). Click here for file

Additional file 7 Allele sizes of six microsatellite loci for 30 individuals representing 19 species of the "ossified group" of lamprologines. Click here for file

Additional file 8 Allele sizes of six microsatellite loci in four hybrid specimens collected at Wonzye and characterization of the genetic diversity in the parental species at the same locality, with allele size range, expected (H_e_) and observed (H_o_) heterozygosity. Click here for file
