# Supplementary material for: Reticulate phylogeny of gastropod-shell-breeding cichlids from Lake Tanganyika – the result of repeated introgressive hybridization
Source: BMC Evol Biol. 2007 Jan 25;7:7. doi: 10.1186/1471-2148-7-7 (PMC1790888; doi:10.1186/1471-2148-7-7)
Supplement: Additional file 1 — Species information, geographic origin and GenBank accession numbers of all taxa used for phylogenetic analysis. [file 1471-2148-7-7-S1.doc]

### Additional File 1 - Species information, sample ID, geographic origin and GenBank accession numbers for all taxa used for phylogenetic analysis.

| **Species** | **ID** | **Locality** | **Coordinates** | **GenBank Acc.Nr. ND2** | **AFLPs** |
| --- | --- | --- | --- | --- | --- |
| *Variabilichromis moorii* | 1508 | Kachese | S 08° 29’, E 30° 28’ | DQ055016 | + |
| *Telmatochromis vittatus* | 2119 | Mtondwe Island | S 08° 42’, E 31° 07’ | AY682545 | - |
|  | 4244 | Wonzye | S 08° 43’, E 31° 08’ | - | + |
| *Julidochromis ornatus* | 2192 | Kasakalawe | S 08° 47’, E 31° 04’ | EF191082 | + |
| *Altolamprologus calvus* | 2481 | Nakaku | S 08° 40’, E 30° 54’ | EF191108 | - |
|  | 3899 | Kapembwa | S 08° 37’, E 30° 51’ | - | + |
|  | 4099 | Chaitika | S 08° 34’, E 30° 47’ | - | + |
| *Altolamprologus compressiceps* | 2482 | Nakaku | S 08° 40’, E 30° 54’ | EF191105 | - |
|  | 3122 | Kigoma* | S 04° 52’, E 29° 37’ | EF191121 | - |
|  | 3900 | Kalambo Lodge | S 08° 37’, E 31° 37’ | - | + |
|  | 3988 | Kalambo | S 08° 37’, E 31° 11’ | - | + |
| *Altolamprologus* sp. „shell“ | 1723 | ?* | ? | AF398229 | - |
|  | 586 | Sumbu | S 08° 31’, E 30° 29’ | EF191107 | - |
|  | 4106 | ?* | ? | - | + |
| *Lamprologus callipterus* | 577 | Mpulungu | S 08° 46’, E 31° 06’ | AF398226 | - |
|  | 1724 | Sondwa | S 08° 44’, E 31° 08’ | DQ055023 | - |
|  | 2517 | Wonzye | S 08° 43’, E 31° 08’ | EF191085 | - |
|  | 4263 | Wonzye | S 08° 43’, E 31° 08’ | - | + |
|  | 4270 | Wonzye | S 08° 43’, E 31° 08’ | - | + |
|  | 4284 | Wonzye | S 08° 43’, E 31° 08’ | - | + |
| *Lamprologus kungweensis* | 2521 | ?* | ? | EF191084 | - |
| *Lamprologus laparogramma* | 579 | Mpulungu | S 08° 46’, E 31° 06’ | EF191087 | - |
|  | 1836 | Mbita Island | S 08° 45’, E 31° 05’ | EF191088 | - |
| *Lamprologus lemairii* | 582 | Mpulungu | S 08° 46’, E 31° 06’ | AY7403786 | - |
|  | 1715 | Mpulungu | S 08° 46’, E 31° 06’ | DQ055019 | - |
|  | 3124 | Kasakalawe | S 08° 47’, E 31° 04’ | EF191093 | - |
|  | 3904 | Mpulungu | S 08° 46’, E 31° 06’ | - | + |
|  | 4100 | Kalambo Lodge | S 08° 37’, E 31° 37’ | - | + |
| *Lamprologus meleagris* | 1733 | Kalubamba | S 07° 23’, E 30° 11’ | DQ055027 | + |
|  | 1734 | Kalubamba | S 07° 23’, E 30° 11’ | EF191097 | - |
|  | 1912 | ?* | ? | EF191098 | + |
| *Lamprologus ocellatus* | 1833 | Chisansa | S 08° 40’, E 31° 11’ | EF191113 | + |
|  | 2199 | Wonzye | S 08° 43’, E 31° 08’ | EF191114 | - |
|  | 2200 | Wonzye | S 08° 43’, E 31° 08’ | EF191115 | - |
|  | 3925 | Kasakalawe | S 08° 47’, E 31° 04’ | - | + |
| *Lamprologus ornatipinnis* | 573 | Mpulungu | S 08° 46’, E 31° 06’ | EF191109 | - |
|  | 1579 | Mtondwe Island | S 08° 42’, E 31° 07’ | EF191110 | + |
|  | 1903 | ?* | ? | EF191111 | - |
|  | 1904 | ?* | ? | EF191112 | + |
| *Lamprologus signatus* | 578 | Mpulungu | S 08° 46’, E 31° 06’ | EF191086 | - |
|  | 1910 | ?* | ? | - | + |
| *Lamprologus speciosus* | 1743 | Masanza | S 07° 33’, E 30° 13’ | DQ055032 | - |
|  | 1744 | Masanza | S 07° 33’, E 30° 13’ | EF191101 | - |
|  | 1908 | ?* | ? | EF191102 | + |
|  | 14 | ?* | ? | - | + |
| *Lepidiolamprologus attenuatus* | 1827 | Mtondwe Island | S 08° 42’, E 31° 07’ | DQ055036 |  |
|  | 1828 | Mpulungu | S 08° 46’, E 31° 06’ | DQ055037 |  |
|  | 2695 | Wonzye | S 08° 43’, E 31° 08’ | DQ055057 |  |
|  | 3906 | Mtondwe Island | S 08° 42’, E 31° 07’ | - | + |
| *Lepidiolamprologus boulengeri* | 1748 | ?* | ? | DQ055034 | - |
|  | 1909 | ?* | ? | DQ055040 | - |
| *Lepidiolamprologus elongatus* | 1450 | Chituta Bay | S 08° 44’, E 31° 09’ | EF191092 | - |
|  | 3909 | Katoto | S 08° 48’, E 31° 01’ | - | + |
|  | 3982 | Kasakalawe | S 08° 47’, E 31° 04’ | - | + |
| *Lepidiolamprologus hecqui* | 1707 | ?* | ? | DQ055018 | - |
|  | 1913 | ?* | ? | DQ055041 | + |
| *Lepidiolamprologus meeli* | 2479 | Kigoma* | S 04° 52’, E 29° 37’ | DQ055051 | + |
| *Lepidiolamprologus profundicola* | 1729 | ?* | ? | DQ055025 | - |
|  | 4107 | Chituta Bay | S 08° 44’, E 31° 09’ | - | + |
| *Lepdiolamprologus* sp.”meeli-boulengeri” | 1831 | Mbita Island | S 08° 45’, E 31° 05’ | DQ055038 | + |
|  | 2511 | Mbita Island | S 08° 45’, E 31° 05’ | DQ055052 | + |
| *Lepidiolamprologus* sp. nov. | 3923 | Katoto | S 08° 48’, E 31° 01’ | - | + |
| *Neolamprologus brevis* | 575 | Mpulungu | S 08° 46’, E 31° 06’ | EF191094 | - |
|  | 1730 | ?* | ? | EF191095 | - |
|  | 4247 | Kigoma* | S 04° 52’, E 29° 37’ | - | + |
|  | 4252 | Kigoma* | S 04° 52’, E 29° 37’ | - | + |
| *Neolamprologus calliurus* | 576 | Mpulungu | S 08° 46’, E 31° 06’ | EF191083 | - |
|  | 2522 | ?* | ? | EF191117 | - |
|  | 2198 | Wonzye | S 08° 43’, E 31° 08’ | EF191096 | - |
|  | 4221 | Wonzye | S 08° 43’, E 31° 08’ | - | + |
|  | 4260 | Wonzye | S 08° 43’, E 31° 08’ | - | + |
|  | 4266 | Wonzye | S 08° 43’, E 31° 08’ | - | + |
| *Neolamprologus caudopunctatus* | 208 | ?* | ? | AY740388 | - |
|  | 1728 | ?* | ? | DQ055024 | - |
|  | 2987 | Nakaku | S 08° 40’, E 30° 54’ | EF191122 | - |
|  | 3910 | Kalambo | S 08° 37’, E 31° 11’ | - | + |
| *Neolamprologus fasciatus* | 1712 | Sondwa | S 08° 44’, E 31° 08’ | EF191119 | - |
|  | 2516 | Wonzye | S 08° 43’, E 31° 08’ | EF191120 | - |
|  | 4233 | Wonzye | S 08° 43’, E 31° 08’ | - | + |
|  | 4238 | Wonzye | S 08° 43’, E 31° 08’ | - | + |
|  | 4249 | Wonzye | S 08° 43’, E 31° 08’ | - | + |
| *Neolamprologus leloupi* | 1906 | ?* | ? | EF191103 | + |
|  | 1907 | ?* | ? | EF191104 | - |
| *Neolamprologus multifasciatus* | 310 | ?* | ? | EF191089 | - |
|  | 580 | Mpulungu | S 08° 46’, E 31° 06’ | EF191090 | - |
|  | 2480 | Mbita Island | S 08° 45’, E 31° 05’ | EF191091 | - |
|  | 3916 | Mbita Island | S 08° 45’, E 31° 05’ | - | + |
|  | 3 | ?* | ? | - | + |
| *Neolamprologus similis* | 1739 | Tembwe | S 07° 14’, E 30° 07’ | DQ055030 | - |
|  | 1740 | Tembwe | S 07° 14’, E 30° 07’ | EF191099 | - |
|  | 1902 | ?* | ? | EF191100 | + |
| *Neolamprologus wauthioni* | 2518 | ?* | ? | EF191116 | + |
|  | 2864 | ?* | ? | EF191118 | + |
|  | 8T00 | ?* | ? | - | + |
| Hybrid 1 | 2566 | Wonzye | S 08° 43’, E 31° 08’ | EF191124 | - |
|  | 2567 | Wonzye | S 08° 43’, E 31° 08’ | EF191125 | - |
| Hybrid 2 | 2565 | Wonzye | S 08° 43’, E 31° 08’ | EF191123 | - |
|  | 3863 | Wonzye | S 08° 43’, E 31° 08’ | EF191126 | - |

*Note:* * samples obtained from aquarium trade.
