# Supplementary material for: Reticulate phylogeny of gastropod-shell-breeding cichlids from Lake Tanganyika – the result of repeated introgressive hybridization
Source: BMC Evol Biol. 2007 Jan 25;7:7. doi: 10.1186/1471-2148-7-7 (PMC1790888; doi:10.1186/1471-2148-7-7)
Supplement: Additional file 2 — Matrix of characters used for estimating similarity indices between hybrids and candidate parental species. [file 1471-2148-7-7-S2.doc]

**Additional File 2 -** Matrix of characters used for estimating similarity indices between hybrids and candidate parental species.

| **Species** | | **Character number** | | | | | | | | | | | | |
| --- | --- | --- | --- | --- | --- | --- | --- | --- | --- | --- | --- | --- | --- | --- |
|  |  | **1** | **2** | **3** | **4** | **5** | **6** | **7** | **8** | **9** | **10** | **11** | **12** | **13** |
| A | Hybrid 1 | 0 | 0 | 0 | 1 | 0 | 0 | 1 | 1 | 0 | 0 | 0 | 1 | 0 |
| B | Hybrid 2 | 1 | 0 | 0 | 0 | 0 | 1 | 0 | 0 | 0 | 0 | 0 | 1 | 0 |
| C | *L. callipterus* | 0 | 0 | 0 | 1 | 0 | 0 | 1 | 1 | 0 | 0 | 0 | 0 | 0 |
| D | *L. lemairii* | 0 | 0 | 0 | 0 | 0 | 0 | 0 | 1 | 0 | 1 | 0 | 0 | 0 |
| E | *L. attenuatus* | 1 | 0 | 0 | 1 | 0 | 0 | 1 | 1 | 1 | 0 | 0 | 1 | 0 |
| F | *N. brevis/calliurus* | 0 | 0 | 0 | 1 | 0 | 0 | 1 | 1 | 0 | 0 | 0 | 1 | 0 |
| G | *N. caudopunctatus* | 0 | 0 | 0 | 1 | 0 | 0 | 1 | 1 | 1 | 0 | 0 | 1 | 0 |
| H | *N. fasciatus* | 1 | 0 | 0 | 1 | 1 | 1 | 1 | 0 | 1 | 0 | 0 | 1 | 0 |
| I | *T. temporalis* | 0 | 0 | 0 | 1 | 0 | 0 | 0 | 0 | 0 | 1 | 1 | 1 | 0 |
| J | *T. vittatus* | 0 | 1 | 1 | 0 | 1 | 1 | 0 | 0 | 0 | 1 | 1 | 1 | 0 |

*Notes:* 1, horizontal bands on lateral body side; 2, lateral stripe on body; 3, dorsal medial band on body; 4, horizontal bands on caudal fin; 5, stripe on dorsal side of snout; 6, blotch on the basis of caudal fin; 7, scales on dorsal head surface; 8, scales on operculum; 9, scales on chest; 10, snout isognathous; 11, caudal end of operculum pointed; 12, outer soft ray of pelvic fin longest; 13, tricuspid teeth on jaws.
