# Supplementary material for: Reticulate phylogeny of gastropod-shell-breeding cichlids from Lake Tanganyika – the result of repeated introgressive hybridization
Source: BMC Evol Biol. 2007 Jan 25;7:7. doi: 10.1186/1471-2148-7-7 (PMC1790888; doi:10.1186/1471-2148-7-7)
Supplement: Additional file 3 — Morphometric measurements for PCA of hybrids and parental species. [file 1471-2148-7-7-S3.doc]

**Additional File 3 -** Morphometric measurements for PCA of hybrids and parental species (cm).

|  | **HUMZ** | **SL** | **BD** | **HL** | **SnL** | **EyL** | **IOW** | **ULL** | **LJL** | **CPL** | **CPD** | **PrDL** | **DBsL** | **ABsL** |
| --- | --- | --- | --- | --- | --- | --- | --- | --- | --- | --- | --- | --- | --- | --- |
| Hybrid 1.1 | 183755 | 39.4 | 10.2 | 13.1 | 3.2 | 4.0 | 3.0 | 5.1 | 6.8 | 6.5 | 4.9 | 12.9 | 22.0 | 10.7 |
| Hybrid 1.2 | 184364 | 38.4 | 10.6 | 13.3 | 4.0 | 4.1 | 2.9 | 5.2 | 6.8 | 6.5 | 4.9 | 13.0 | 21.6 | 10.3 |
| Hybrid 2.1 | 183808 | 72.2 | 21.4 | 22.9 | 8.0 | 6.4 | 6.1 | 9.8 | 11.5 | 12.4 | 9.0 | 22.4 | 40.5 | 21.3 |
| Hybrid 2.2 | 183772 | 73.4 | 21.4 | 23.0 | 7.5 | 6.0 | 6.5 | 10.3 | 11.5 | 13.0 | 9.4 | 21.8 | 40.9 | 20.9 |
| *N. brevis* | 174045 | 51.1 | 14.9 | 16.0 | 4.9 | 4.8 | 4.2 | 6.7 | 7.7 | 8.4 | 7.0 | 14.9 | 31.4 | 14.0 |
| *N. brevis* | 174046 | 45.9 | 13.6 | 15.0 | 4.8 | 4.5 | 4.1 | 7.1 | 7.6 | 8.0 | 7.0 | 14.5 | 26.2 | 11.6 |
| *N. brevis* | 174047 | 42.1 | 14.0 | 14.5 | 4.4 | 4.4 | 3.9 | 6.3 | 7.2 | 7.2 | 6.6 | 13.0 | 24.5 | 11.2 |
| *N. brevis* | 174049 | 43.5 | 14.1 | 15.0 | 4.5 | 4.4 | 4.0 | 7.0 | 7.9 | 7.9 | 6.6 | 13.0 | 24.0 | 12.2 |
| *N. brevis* | 174050 | 46.9 | 14.5 | 14.9 | 4.5 | 4.6 | 4.5 | 6.5 | 7.5 | 8.1 | 7.0 | 13.4 | 28.5 | 14.2 |
| *L. callipterus* | 173736 | 83.8 | 23.7 | 27.2 | 9.8 | 6.5 | 6.7 | 10.9 | 13.1 | 14.6 | 9.6 | 28.1 | 47.7 | 22.8 |
| *L. callipterus* | 173758 | 94.5 | 26.9 | 30.6 | 11.5 | 6.7 | 8.2 | 12.5 | 14.7 | 15.8 | 10.4 | 30.4 | 51.5 | 26.5 |
| *L. callipterus* | 176422 | 71.9 | 19.5 | 22.0 | 8.0 | 8.9 | 5.8 | 9.5 | 11.2 | 11.6 | 7.9 | 22.4 | 38.7 | 20.0 |
| *L. callipterus* | 175987 | 78.6 | 22.0 | 25.1 | 9.2 | 6.5 | 6.2 | 9.9 | 11.8 | 14.0 | 8.5 | 25.7 | 44.1 | 21.2 |
| *L. callipterus* | 176473 | 66.5 | 19.2 | 22.6 | 7.9 | 6.0 | 6.5 | 8.8 | 10.4 | 12.0 | 7.5 | 21.4 | 35.9 | 18.8 |
| *N. fasciatus* | 174245 | 65.5 | 18.0 | 24.3 | 8.0 | 7.0 | 4.3 | 9.5 | 11.5 | 9.2 | 7.2 | 23.0 | 35.0 | 18.1 |
| *N. fasciatus* | 175985 | 80.6 | 20.8 | 29.2 | 10.2 | 7.5 | 5.6 | 11.0 | 15.9 | 14.0 | 9.3 | 29.2 | 46.0 | 20.9 |
| *N. fasciatus* | 176418 | 83.3 | 20.5 | 28.5 | 10.5 | 7.5 | 5.5 | 9.2 | 14.7 | 15.9 | 8.9 | 28.0 | 44.9 | 20.0 |
| *N. fasciatus* | 176476 | 78.4 | 18.5 | 28.4 | 9.6 | 7.5 | 5.5 | 11.1 | 15.0 | 12.8 | 8.3 | 26.2 | 41.4 | 22.6 |
| *N. fasciatus* | 176666 | 99.8 | 24.7 | 35.9 | 14.2 | 8.6 | 6.2 | 11.2 | 19.2 | 15.9 | 10.4 | 34.2 | 54.8 | 28.0 |

*Notes:* HUMZ, voucher specimen numbers of the Hokkaido University Laboratory of Marin Zoology, Faculty of Fisheries; SL, standard length; BD, body depth; HL, head length; SnL, snout length; EyL, horizontal diameter of eye; IOW, interorbital width; UJL, upper jaw length; LJL, lower jaw length; CPL, caudal peduncle length; CPD, caudal peduncle depth; PrDL, predorsal length; ABsL, anal base length.
