# Supplementary material for: Reticulate phylogeny of gastropod-shell-breeding cichlids from Lake Tanganyika – the result of repeated introgressive hybridization
Source: BMC Evol Biol. 2007 Jan 25;7:7. doi: 10.1186/1471-2148-7-7 (PMC1790888; doi:10.1186/1471-2148-7-7)
Supplement: Additional file 4 — Meristic measurements for PCA of hybrids and parental species. [file 1471-2148-7-7-S4.doc]

**Additional File 4 -** Meristic measurements for PCA of hybrids and parental species.

|  | **HUMZ** | **Ds** | **Dr** | **As** | **Ar** | **ScLL** | **ScULL** | **ScLLL** | **GR** |
| --- | --- | --- | --- | --- | --- | --- | --- | --- | --- |
| Hybrid 1.1 | 183755 | 19 | 9 | 9 | 7 | 36 | 28 | 15 | 16 |
| Hybrid 1.2 | 184364 | 19 | 9 | 8 | 7 | 35 | 27 | 13 | 16 |
| Hybrid 2.1 | 183808 | 19 | 9 | 9 | 6 | 36 | 26 | 9 | 15 |
| Hybrid 2.2 | 183772 | 19 | 8 | 9 | 6 | 34 | 23 | 6 | 13 |
| *N. brevis/calliurus* | 174045 | 19 | 9 | 9 | 7 | 34 | 23 | 8 | 20 |
| *N. brevis/calliurus* | 174046 | 19 | 8 | 8 | 6 | 35 | 19 | 0 | 18 |
| *N. brevis/calliurus* | 174047 | 18 | 9 | 8 | 7 | 33 | 29 | 10 | 20 |
| *N. brevis/calliurus* | 174049 | 19 | 8 | 8 | 7 | 34 | 16 | 0 | 19 |
| *N. brevis/calliurus* | 174050 | 20 | 7 | 9 | 6 | 35 | 26 | 6 | 20 |
| *L. callipterus* | 173736 | 19 | 9 | 8 | 8 | 37 | 27 | 16 | 11 |
| *L. callipterus* | 173758 | 19 | 9 | 8 | 8 | 36 | 26 | 14 | 11 |
| *L. callipterus* | 176422 | 19 | 9 | 8 | 8 | 35 | 25 | 13 | 13 |
| *L. callipterus* | 175987 | 18 | 9 | 8 | 8 | 36 | 26 | 15 | 13 |
| *L. callipterus* | 176473 | 19 | 10 | 8 | 8 | 38 | 26 | 11 | 13 |
| *N. fasciatus* | 174245 | 19 | 9 | 10 | 7 | 33 | 25 | 10 | 9 |
| *N. fasciatus* | 175985 | 19 | 9 | 10 | 6 | 33 | 25 | 13 | 11 |
| *N. fasciatus* | 176418 | 19 | 9 | 9 | 7 | 34 | 23 | 10 | 12 |
| *N. fasciatus* | 176476 | 19 | 9 | 10 | 7 | 33 | 23 | 16 | 12 |
| *N. fasciatus* | 176666 | 18 | 10 | 10 | 7 | 37 | 22 | 12 | 12 |

*Notes:* HUMZ, voucher specimen numbers of the Hokkaido University Laboratory of Marin Zoology, Faculty of Fisheries; Ds, dorsal fin spines; Dr, dorsal fin soft rays; As, anal fin spines; Ar, anal fin soft rays; ScLL, scales on longitudinal line; ScULL, scales on upper lateral line; ScLLL, scales on lower lateral line; GR, gill rakers on lower limb of first gill arch.
