# Supplementary material for: Reticulate phylogeny of gastropod-shell-breeding cichlids from Lake Tanganyika – the result of repeated introgressive hybridization
Source: BMC Evol Biol. 2007 Jan 25;7:7. doi: 10.1186/1471-2148-7-7 (PMC1790888; doi:10.1186/1471-2148-7-7)
Supplement: Additional file 5 — Factor loadings of morphometric measurements on the first three principal components of Lamprologus callipterus (n = 5), Neolamprologus brevis (n = 5), N. fasciatus (n = 5), hybrid 1 (n = 2) and hybrid 2 (n = 2). [file 1471-2148-7-7-S5.doc]

**Additional File 5 -** Factor loadings of morphometric measurements on the first three principal components of *Lamprologus callipterus* (n=5), *Neolamprologus brevis/calliurus* (n=5), *N. fasciatus* (n=5), hybrid 1 (n=2) and hybrid 2 (n=2).

|  | **PC1** | **PC2** | **PC3** |
| --- | --- | --- | --- |
| Standard length | 0.9996 | -0.0088 | 0.0051 |
| Body depth | 0.9572 | -0.2553 | 0.0375 |
| Head length | 0.9817 | 0.1754 | 0.0304 |
| Snout length | 0.9828 | 0.1417 | 0.0030 |
| EyL | 0.8634 | 0.2004 | 0.1572 |
| IOW | 0.8683 | -0.4260 | 0.1020 |
| ULL | 0.9504 | -0.0799 | 0.1806 |
| LJL | 0.9536 | 0.2687 | 0.0091 |
| CPL | 0.9724 | -0.0733 | -0.1321 |
| CPD | 0.9374 | -0.2207 | -0.0418 |
| PrDL | 0.9872 | 0.1368 | -0.0293 |
| DBsL | 0.9943 | -0.0600 | -0.0639 |
| ABsL | 0.9826 | -0.0762 | 0.1422 |
| Proportion of overall variation | 0.9833 | 0.0092 | 0.0024 |
