# Supplementary material for: Reticulate phylogeny of gastropod-shell-breeding cichlids from Lake Tanganyika – the result of repeated introgressive hybridization
Source: BMC Evol Biol. 2007 Jan 25;7:7. doi: 10.1186/1471-2148-7-7 (PMC1790888; doi:10.1186/1471-2148-7-7)
Supplement: Additional file 6 — Factor loadings of meristic measurements on the first three principal components of Lamprologus callipterus (n = 5), Neolamprologus brevis (n = 5), N. fasciatus (n = 5), hybrid 1 (n = 2) and hybrid 2 (n = 2). [file 1471-2148-7-7-S6.doc]

**Additional File 6 - Factor loadings of meristic measurements on the first three principal components of *Lamprologus callipterus* (n=5), *Neolamprologus brevis/calliurus* (n=5), *N. fasciatus* (n=5), hybrid 1 (n=2) and hybrid 2 (n=2).**

|  | **PC1** | **PC2** |
| --- | --- | --- |
| Ds | -0.2365 | 0.0225 |
| Dr | 0.6224 | -0.1576 |
| As | 0.1715 | -0.3598 |
| Ar | 0.5304 | -0.0287 |
| ScLL | 0.2619 | 0.0609 |
| ScULL | 0.7271 | 0.5900 |
| ScLLL | 0.9794 | 0.0836 |
| GR | -0.6879 | 0.6988 |
| Proportion of overall variation | 0.6788 | 0.1988 |
