# Supplementary material for: Reticulate phylogeny of gastropod-shell-breeding cichlids from Lake Tanganyika – the result of repeated introgressive hybridization
Source: BMC Evol Biol. 2007 Jan 25;7:7. doi: 10.1186/1471-2148-7-7 (PMC1790888; doi:10.1186/1471-2148-7-7)
Supplement: Additional file 7 — Allele sizes of six microsatellite loci for 30 individuals representing 19 species of the "ossified group" of lamprologines. [file 1471-2148-7-7-S7.doc]

**Additional File 7 -** Allele sizes (bp) of six microsatellite loci for 30 individuals representing 19 species of “ossified group lamprologines”.

| **Species** | **Locality** | **Locus** | | | | | | | | | | | |
| --- | --- | --- | --- | --- | --- | --- | --- | --- | --- | --- | --- | --- | --- |
|  |  | **Pzeb3** | | **TmoM25** | | **TmoM27** | | **UNH154** | | **UNH855** | | **UNH952** | |
| *N. similis* | Tembwe | - | - | 355 | 355 | 374 | 374 | 88 | 88 | - | - | 155 | 155 |
| *N. similis* | Tembwe | - | - | 355 | 355 | 374 | 374 | 88 | 88 | - | - | 155 | 155 |
| *N. similis* | ?* | 315 | 315 | 355 | 355 | 374 | 374 | 88 | 88 | 151 | 153 | 155 | 155 |
| *L. signatus* | Mpulungu | 295 | 295 | 367 | 373 | 382 | 382 | 100 | 100 | 149 | 149 | 133 | 145 |
| *L. ornatipinnis* | Mpulungu | 321 | 321 | 373 | 373 | 382 | 382 | 94 | 94 | 149 | 149 | 155 | 155 |
| *N. multifasciatus* | Mpulungu | 321 | 321 | 363 | 363 | 372 | 372 | 88 | 88 | 149 | 149 | 141 | 155 |
| *N. multifasciatus* | Mbita Island | 321 | 323 | 363 | 363 | 372 | 372 | 88 | 88 | 149 | 149 | 139 | 155 |
| *N. calliurus* | Mpulungu | 309 | 311 | 345 | 361 | 382 | 384 | 90 | 96 | 151 | 153 | 131 | 135 |
| *N. calliurus* | ?* | 309 | 309 | 361 | 361 | 384 | 384 | 94 | 116 | 153 | 153 | 135 | 135 |
| *L. speciosus* | Masanza | 311 | 311 | 361 | 361 | 378 | 378 | 90 | 90 | 149 | 149 | 159 | 159 |
| *L. speciosus* | Masanza | 311 | 311 | 361 | 361 | 378 | 378 | 90 | 90 | 149 | 149 | 159 | 159 |
| *L. speciosus* | ?* | 311 | 311 | 361 | 361 | 378 | 378 | 90 | 90 | 149 | 149 | 159 | 159 |
| *L. meleagris* | Kalubamba | 311 | 311 | 341 | 341 | 378 | 378 | 110 | 110 | 147 | 147 | 133 | 133 |
| *L. meleagris* | Kalubamba | 311 | 311 | 341 | 341 | 378 | 378 | 110 | 110 | 147 | 147 | 133 | 133 |
| *L. meleagris* | ?* | 311 | 311 | 341 | 341 | 378 | 378 | 110 | 110 | 147 | 147 | 133 | 133 |
| *N. caudopunctatus* | Nakaku | 315 | 315 | 373 | 379 | 374 | 374 | 92 | 92 | 149 | 149 | 145 | 145 |
| *L. elongatus* | Kalambo Lodge | 315 | 315 | 361 | 361 | 372 | 372 | 88 | 94 | 149 | 149 | 135 | 135 |
| *L. profundicola* | Chituta Bay | 315 | 315 | 361 | 361 | 372 | 372 | 90 | 90 | 149 | 149 | 143 | 143 |
| *L. hecqui* | ?* | 313 | 313 | 361 | 361 | 372 | 372 | 90 | 90 | 149 | 149 | 135 | 141 |
| *L. meeli* | Kigoma* | 311 | 311 | 367 | 367 | 372 | 372 | 88 | 88 | 149 | 149 | 143 | 145 |
| *L. attenuatus* | Wonzye | 315 | 315 | 365 | 365 | 374 | 382 | 90 | 90 | 149 | 149 | 135 | 143 |
| *L. ocellatus* | Chisansa | 311 | 311 | 355 | 355 | 378 | 378 | 98 | 106 | 149 | 149 | 157 | 157 |
| *L. ocellatus* | Wonzye | 311 | 311 | 355 | 355 | 378 | 378 | 90 | 102 | 149 | 149 | 157 | 157 |
| *L. ocellatus* | Wonzye | 311 | 311 | 355 | 355 | 378 | 378 | 98 | 106 | 149 | 149 | 157 | 159 |
| *A. compressiceps* | Kigoma* | 308 | 316 | 365 | 365 | 382 | 382 | 94 | 100 | 149 | 151 | 143 | 151 |
| *L. callipterus* | Mpulungu | 313 | 319 | 361 | 361 | 376 | 376 | 90 | 90 | 149 | 149 | 151 | 153 |
| *N. wauthioni* | ?* | 311 | 311 | 361 | 361 | 378 | 378 | 90 | 96 | 149 | 149 | 159 | 159 |
| *N. wauthioni* | ?* | 311 | 311 | 361 | 361 | 378 | 378 | 90 | 96 | 149 | 149 | 159 | 159 |
| *N. fasciatus* | ?* | 311 | 311 | 367 | 367 | 370 | 370 | 88 | 88 | 149 | 149 | 161 | 161 |
| *N. fasciatus* | ?* | 311 | 311 | 367 | 367 | 370 | 370 | 88 | 94 | 149 | 149 | 159 | 161 |

*Notes:* Results from population samples of *L. callipterus*, *N. brevis*/*calliurus*, and *N. fasciatus* from Wonzye are listed in Table 3.

* samples obtained from aquarium trade.
