# Supplementary material for: Reticulate phylogeny of gastropod-shell-breeding cichlids from Lake Tanganyika – the result of repeated introgressive hybridization
Source: BMC Evol Biol. 2007 Jan 25;7:7. doi: 10.1186/1471-2148-7-7 (PMC1790888; doi:10.1186/1471-2148-7-7)
Supplement: Additional file 8 — Allele sizes of six microsatellite loci in four hybrid specimens collected at Wonzye and characterization of the genetic diversity in the parental species at the same locality, with allele size range, expected (He) and observed (Ho) heterozygosity. [file 1471-2148-7-7-S8.doc]

**Additional File 8 -** Allele sizes (bp) at six microsatellite loci in four hybrid specimens collected at Wonzye and characterization of the genetic diversity in the parental species at the same locality, with allele size range, expected (H_e_) and observed (H_o_) heterozygosity.

|  |  | **Locus** | | | | | | | | | | | |
| --- | --- | --- | --- | --- | --- | --- | --- | --- | --- | --- | --- | --- | --- |
|  |  | **Pzeb3** | | **TmoM25** | | **TmoM27** | | **UNH154** | | **UNH855** | | **UNH952** | |
| Hybrid 1.1. |  | 313 | 319 | 361 | 361 | 372 | 384 | 90 | 100 | 149 | 151 | 135 | 159 |
| Hybrid 1.2 |  | 313 | 319 | 359 | 361 | 372 | 384 | 90 | 114 | 149 | 153 | 135 | 153 |
| Hybrid 2.1 |  | 309 | 311 | 353 | 367 | 370 | 382 | 88 | 114 | 149 | 153 | 135 | 161 |
| Hybrid 2.2 |  | 309 | 311 | 353 | 367 | 370 | 382 | 88 | 114 | 149 | 153 | 135 | 161 |
|  |  |  | |  | |  | |  | |  | |  | |
| *L. callipterus* (n=19) | range | 309-319 | | 351-361 | | 370-376 | | 88-104 | | 149 | | 151-159 | |
|  | H_e_ | 0.7645 | | 0.6814 | | 0.6219 | | 0.8504 | | 0.0000 | | 583102 | |
|  | H_o_ | 0.6842 | | 0.7368 | | 0.2105 | | 0.8421 | | 0.0000 | | 421053 | |
|  |  |  | |  | |  | |  | |  | |  | |
| *N. brevis/calliurus* (n=24) | range | 309-345 | | 343-371 | | 382-384 | | 94-126 | | 147-153 | | 131-135 | |
|  | H_e_ | 0.6059 | | 0.8620 | | 0.4783 | | 0.8924 | | 0.6102 | | 0.1918 | |
|  | H_o_ | 0.5833 | | 0.5833 | | 0.1250 | | 0.5833 | | 0.4167 | | 0.1250 | |
|  |  |  | |  | |  | |  | |  | |  | |
| *N. fasciatus* (n=21) | range | 311 | | 361-367 | | 370 | | 88-102 | | 149 | | 159-161 | |
|  | H_e_ | 0.0000 | | 0.0465 | | 0.0000 | | 0.5499 | | 0.0000 | | 0.0465 | |
|  | H_o_ | 0.0000 | | 0.0476 | | 0.0000 | | 0.4286 | | 0.0000 | | 0.0476 | |
